# Investigating Tumor Heterogeneity in Mouse Models

**DOI:** 10.1146/annurev-cancerbio-030419-033413

**Published:** 2019-11-06

**Authors:** Tuomas Tammela, Julien Sage

**Affiliations:** 1 Cancer Biology and Genetics Program, Memorial Sloan Kettering Cancer Center, New York, NY 10065, USA; 2 Department of Pediatrics and Department of Genetics, Stanford University, Stanford, California 94305, USA

**Keywords:** tumor heterogeneity, mouse models, genetics, epigenetics, tumor microenvironment, cancer stem cells

## Abstract

Cancer arises from a single cell through a series of acquired mutations and epigenetic alterations. Tumors gradually develop into a complex tissue comprised of phenotypically heterogeneous cancer cell populations, as well as noncancer cells that make up the tumor microenvironment. The phenotype, or state, of each cancer and stromal cell is influenced by a plethora of cell-intrinsic and cell-extrinsic factors. The diversity of these cellular states promotes tumor progression, enables metastasis, and poses a challenge for effective cancer treatments. Thus, the identification of strategies for the therapeutic manipulation of tumor heterogeneity would have significant clinical implications. A major barrier in the field is the difficulty in functionally investigating heterogeneity in tumors in cancer patients. Here we review how mouse models of human cancer can be leveraged to interrogate tumor heterogeneity and to help design better therapeutic strategies.

## HETEROGENEITY IS A HALLMARK OF HUMAN TUMORS DURING TUMOR PROGRESSION AND IN RESPONSE TO THERAPY

**Figure 1:**
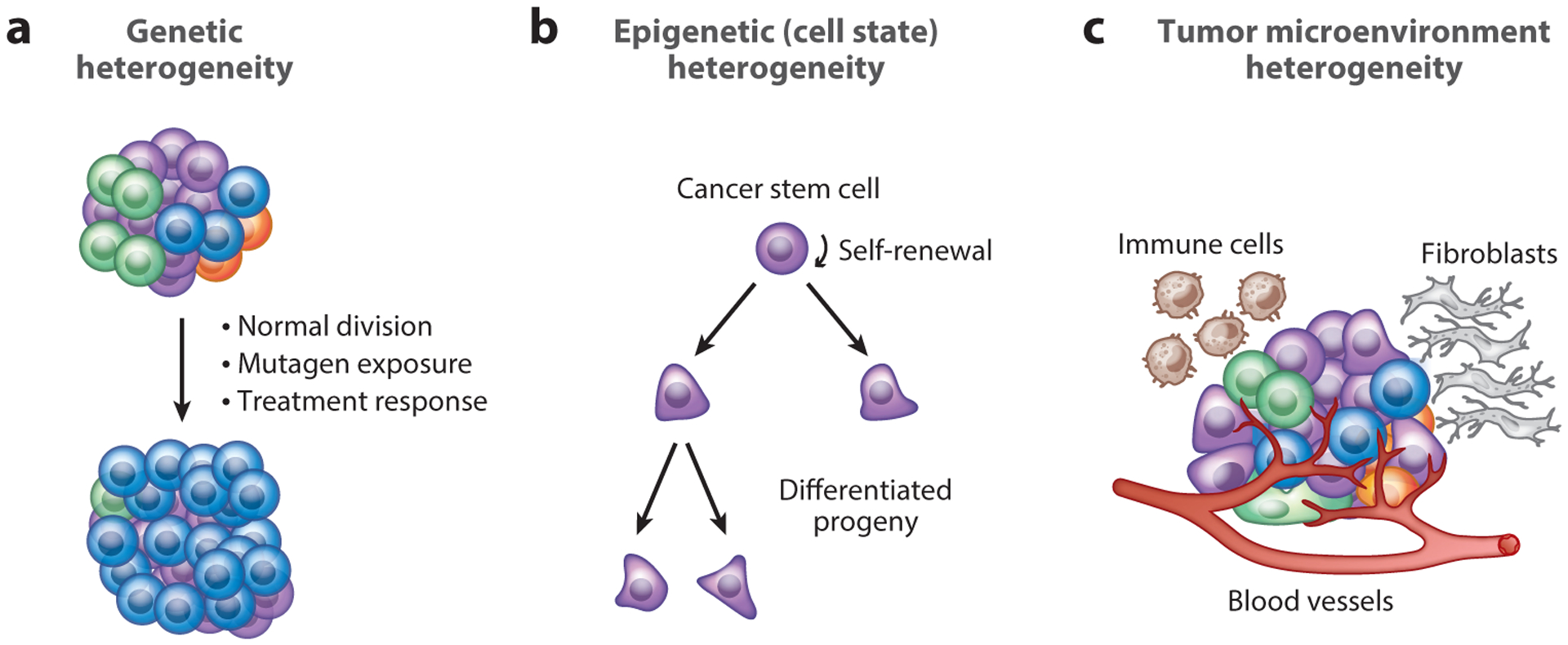
Heterogeneity in cancer. Tumors have several levels of heterogeneity. (*a*) During tumorigenesis, mutations in the DNA and genetic heterogeneity (represented by different colors) can arise from normal cell division, from exposure to mutagens, or in response to treatment in the clinic. This may lead to the appearance (and disappearance) of subclones. (*b*) Even when cancer cells are identical or very similar at the genetic level, tumors are often organized in a hierarchical manner in which cancer stem cells can self-renew and give rise to daughter cells with different transcriptional programs (represented by different shapes). (*c*) Cancer cells in tumors are intimately connected with several noncancer cells; heterogeneity can also be found within each subtype of noncancer cells in the microenvironment (e.g., fibroblasts, endothelial cells, or T cells).

Human tumors develop from single cells in which an accumulation of genetic and epigenetic alterations results in uncontrolled expansion. As cancer cells divide and tumor mass grows, several levels of tissue complexity and heterogeneity emerge (Figure 1).

First, not all cancer cells are genetically identical within a tumor. Cancer cells constantly accumulate genetic differences during normal DNA replication and cell division and also as a direct consequence of environmental mutagens—including some cancer therapies. This genetic heterogeneity exists temporally as tumors expand, spread, and metastasize, but also spatially, with clones and subclones localizing in different areas of a tumor. Genetic heterogeneity has been shown to be a key factor driving tumor evolution and determining response to therapy. An impressive body of literature generated through analysis of human tumors has been reviewed extensively (Ben-David et al. 2019, Dagogo-Jack & Shaw 2018, Hunter et al. 2018, Marusyk & Polyak 2010, McGranahan & Swanton 2017).

Second, cells that are genetically identical (or highly similar) can generate diversity because of epigenetic differences. Changes in DNA methylation or chromatin structure can result in changes in cell fate and differentiation and can profoundly affect the biology of cancer cells. This epigenetic heterogeneity forms the basis for cell state differences between so-called cancer stem cells, which drive the long-term growth of tumors, and their progeny, which are often less capable of long-term expansion due to loss of stem-like features such as self-renewal and extended proliferative capacity (reviewed by Assenov et al. 2018, Easwaran et al. 2014, Marusyk et al. 2012, Shackleton et al. 2009, and Wainwright & Scaffidi 2017). Another integral part of the cancer stem cell model is that tumors generally arise from tissue stem and progenitor cells, which are generally more susceptible to transformation (reviewed by Batlle & Clevers 2017 and Nassar & Blanpain 2016). Features of these cells are propagated in a subpopulation of cancer cells through epigenetic mechanisms as well as through microenvironmental cues (see below) (Lim et al. 2017, Tammela et al. 2017).

Third, tumors are nearly always composed of cancer cells and noncancer cells, including various types of immune cells, fibroblasts, and endothelial cells. Within each group of noncancer cells within the tumor microenvironment, several layers of heterogeneity also exist. This tumor microenvironment heterogeneity controls many aspects of tumor growth, from oxygenation to inflammation and metastasis (reviewed by Balkwill et al. 2012, Fidler et al. 2007, Marusyk & Polyak 2010, and Tredan et al. 2007).

These different aspects of tumor heterogeneity raise several questions: What is the contribution of genetic heterogeneity to phenotypic cell state heterogeneity? Is there a requirement for a core set of cancer cell states independently of the constraints of the genome or epigenome? What is the biological basis of maintaining genetic heterogeneity in cancer? To what extent is epigenetic heterogeneity hardwired in cancer progression? Can intratumoral heterogeneity be targeted therapeutically (or exploited otherwise)?

These questions are often challenging to address in cancer patients because we nearly always lack the ability to investigate human tumors spatially and temporally. Some exceptions include cancers such as breast and colon cancer, in which samples are easier to obtain due to tissue site, or studies like TRACERx [TRAcking Cancer Evolution through therapy (Rx)], in which repeat biopsies are included (Jamal-Hanjani et al. 2014). Still, these rarely reach the level of resolution needed to understand the mechanistic underpinnings of heterogeneity. Circulating tumor cells (CTCs) can provide a way to collect and analyze cancer cells longitudinally (e.g., Hodgkinson et al. 2014), but these CTCs are not in the context of the tumor mass and thus represent a subset of cells that gained access to the circulation. Organoids have reached an impressive level of complexity, including the recent addition of immune cells (e.g., Neal et al. 2018), but it is still unclear whether these ex vivo structures can recapitulate all of the key aspects of tumor development in vivo, including the heterogeneous populations of cancer and stromal cells that functionally interact in primary tumors and metastases.

## MODELING AND STUDYING TUMOR HETEROGENEITY IN GENETICALLY ENGINEERED MICE

### Generating Accurate Mouse Models of Human Cancer

Genetically engineered mouse models (GEMMs) of human disease can help bridge some of the major gaps in human studies. In the past three decades following the development of the first transgenic mice expressing oncogenes, mouse models of human cancer have reached a great level of sophistication and accuracy. The development of inducible models in which oncogenes can be activated and tumor suppressors can be inactivated in a spatially and temporally controlled manner has allowed human cancer development to be modeled very precisely (Frese & Tuveson 2007, Hann & Balmain 2001). While the field has relied extensively in the past 20 years on Cre/Lox models, the more recent development of CRISPR/Cas9 approaches has further accelerated the development of mouse models of human cancers (Sanchez-Rivera & Jacks 2015). These advanced genetic tools now allow investigators to functionally test genetic and genomic alterations identified in human tumors and to examine the interactions between immune cells and cancer cells (reviewed by de Ruiter et al. 2018 and Olson et al. 2018).

Some aspects of human cancer development are difficult to model in mice, including large tumor size or the long period of tumor development (reviewed by Rangarajan & Weinberg 2003), but a major strength of mouse models in this context is the ability to track tumor cells from cancer inception to the development of metastases, as well as in response to therapies. Because the mice used in these experiments live in controlled environments and are genetically similar, tumor development and associated phenotypes are highly reproducible, allowing longitudinal studies that are impossible in humans. Even in cases of repeat biopsies, only a minute fraction of human tumors can be analyzed, while entire mouse tumors are easily obtained.

Another aspect of human cancer development that can be different in mice is the extent of genetic heterogeneity. Even in the most controlled environment, mouse tumors arising from defined genetic events do evolve to be genetically different and unique, similar to human tumors (e.g., McFadden et al. 2014). However, mouse tumors may evolve with a lower level of genetic heterogeneity due to the absence of environmental mutagens in most cases; this low frequency of genetic alterations may be similar to certain human cancers such as pediatric cancers, but it does not recapitulate the high tumor mutation burden (TMB) in human tumors associated with carcinogens such as cigarette smoke or UV light exposure (Chalmers et al. 2017, Grobner et al. 2018). One simple way to mimic the human situation is to treat mice with the same carcinogens that are suspected or known to cause cancer in humans, for example, chemicals found in cigarette smoke (reviewed by Akbay & Kim 2018). Studies combining mouse genetics and treatment with carcinogens have been used, for example, in skin and liver cancer models (e.g., Kastenhuber et al. 2017, McCreery et al. 2015). Another purely genetic approach to increase mutations in mouse tumors would be to cross conditional knockout alleles of genes involved in DNA repair or genome stability into mouse models of cancer [e.g., telomerase (Kwong et al. 2017) or the DNA repair enzyme Mbd4 (Wong et al. 2002)]. Using mutations in genes in the mismatch repair system (e.g., genes coding for Msh2, Msh6, Mlh1, and Pms2) may be useful to model cancers in which these mutations are frequent, such as colorectal cancer (discussed by McIlhatton et al. 2016), but could in theory be combined with any other oncogenic event in mouse models. One possible caveat of studies aiming to bring mouse models closer to human tumors in terms of genetic heterogeneity and higher TMB is that these mouse tumors may then lose key features of what makes mice such an attractive model to study cancer: their controlled genetics and reproducibility. On the other hand, such high-TMB models may help clarify some of the interactions between cancer cells and immune cells, as well as the role of inflammation in cancer.

### Tracking Single Cells and Heterogeneity in Mouse Tumors Temporally and Spatially

Recent exciting developments in the mouse models of human cancer field include the analysis of accurate cancer models in combination with new tools to investigate single-cell biology, a key advance in the study of tumor heterogeneity.

Upon dissection of tumors, flow cytometry has long been used to investigate various subpopulations of cells. However, single-cell RNA sequencing (scRNA-seq) has recently emerged as the contemporary gold standard approach for molecularly defining cell states (or phenotypes) (Tanay & Regev 2017). The sensitivity of current scRNA-seq methods provides quantitative information of 5–15% of polyadenylated transcripts within the cell, enabling the unbiased detection of prominent gene expression programs. These programs, together with individual marker genes, placed in the context of an increasing amount of a priori knowledge about the functional properties of various cancer cell subsets (e.g., stem-like versus differentiated, epithelial versus mesenchymal, proliferative versus quiescent, metastatic versus nonmetastatic), enable the definition of the cancer cell state as well as the identification of new states.

These approaches can be combined or substituted with other methods such as mass cytometry [CyTOF (cytometry by time of flight), e.g., in a mouse model of human prostate cancer (Wang et al. 2016)] and multiplexed spatial cytometry [CODEX (codetection by indexing), e.g., studies in the mouse spleen (Goltsev et al. 2018), and MIBI (multiplexed ion beam imaging by time of flight), e.g., in human breast cancer (Keren et al. 2018)]. Whole tumors can be cleared, labeled, and imaged with various methods [reviewed by Gradinaru et al. 2018; see Hsueh et al. (2017) for an example of clearing human cancer specimens for analysis]. As discussed above, RNA species can be identified and analyzed by scRNA-seq [reviewed by Zhang et al. (2016); e.g., in melanoma (Jerby-Arnon et al. 2018)], but also by spatial transcriptomic arrays (Stahl et al. 2016) and by highly multiplexed in situ hybridization approaches preserving the spatial identity of cells (Eng et al. 2019, Moffitt & Zhuang 2016, Rodriques et al. 2019). The rapidly expanding repertoire of single-cell methods enables cell states to be defined with increasing precision (see https://github.com/arnavm/multimodal-scRNA-seq for a list of these technologies).

An important issue in tumor heterogeneity is to understand mechanisms underlying cell fate determination. To do this, one must be able to connect the cell’s present (transcriptional) state with its ancestry. scRNA-seq, coupled to an expanding set of sophisticated computational methods, provides a platform for inferring clonal relationships based on the similarity (or dissimilarity) of the cells’ transcriptional profiles (Herring et al. 2018, Kester & van Oudenaarden 2018). These computational methods can be used to construct pseudo-time trajectories of cellular differentiation from single samples of dynamic tissues. An elegant example of such an approach is Wishbone, which allows one to identify branch points in a differentiation trajectory (Setty et al. 2016). An extension of these methods, RNA velocity (La Manno et al. 2018), enables one to predict the probability of a cell acquiring a new state by analyzing prespliced transcripts and mature messenger RNAs within single cells. Another method based on inference of temporal coupling using optimal transport theory—an eighteenth century mathematical paradigm—was recently reported (Schiebinger et al. 2019).

Although increasingly powerful, computational inference methods do not provide definitive information on clonal relationships in tissues. Spontaneous somatic mutations enable the retrospective reconstruction of lineage trees in tissues, which has been widely applied to both human and mouse tumors (e.g., McFadden et al. 2014, Morrissy et al. 2017, Turajlic et al. 2018). Recently reported computational approaches utilizing, e.g., rapidly mutating mitochondrial DNA (Ludwig et al. 2019) or varying lengths of polyguanine repeats (Naxerova et al. 2017) from scRNA-seq or whole-genome sequencing data, respectively, are better suited for single-cell studies where sequencing depth has been an issue. However, these approaches lack the ability to prospectively probe cell fate determination of a subpopulation of interest, which is readily doable in GEMMs.

**Figure 2:**
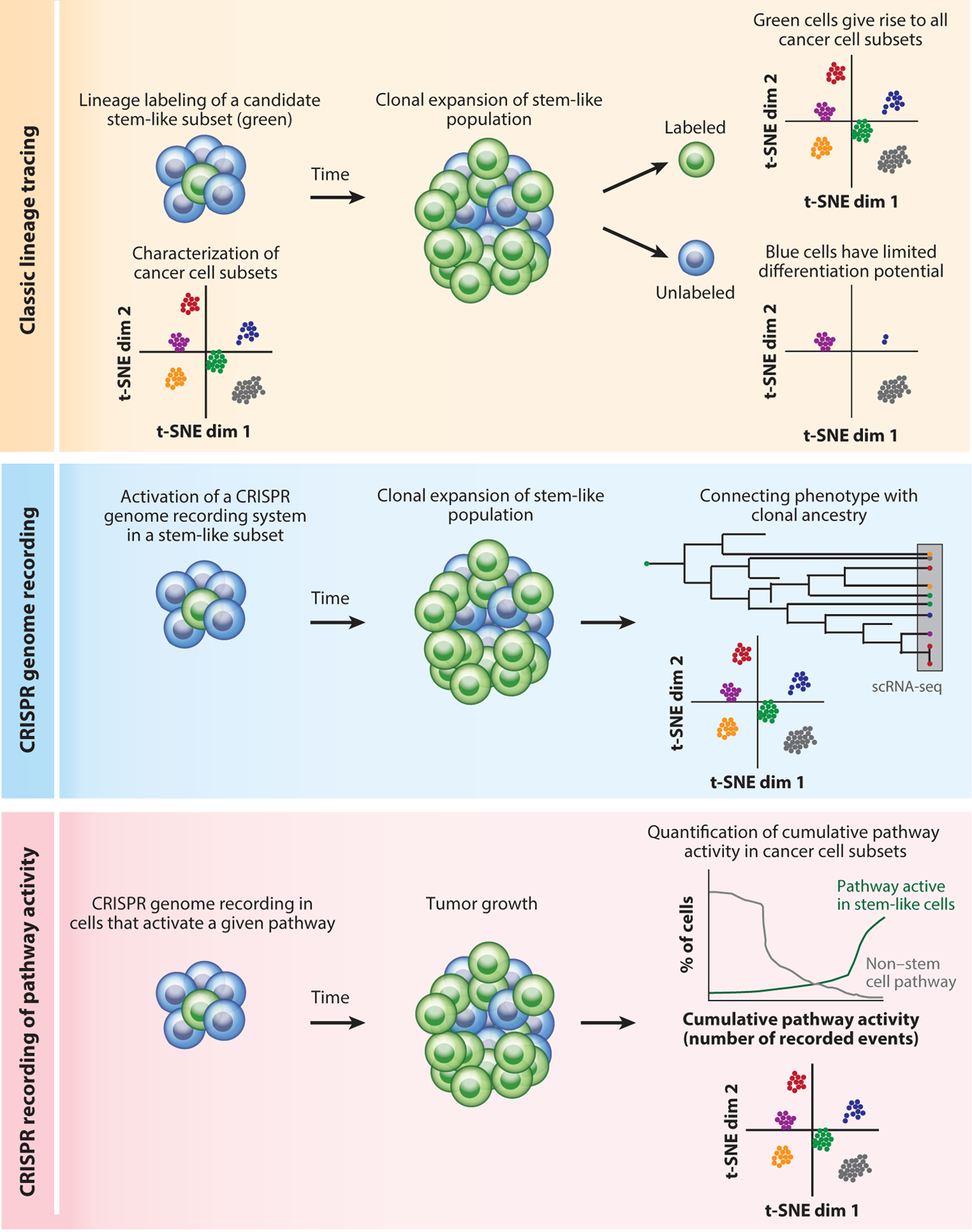
Lineage-tracing strategies for the prospective interrogation of cell fate determination in cancer. (*Top*) Classic lineage tracing is based on the introduction of a heritable genetic mark, e.g., a fluorescent protein (*green*). Cancer cells with stem-like properties marked in this way will clonally expand upon tumor growth. scRNA-seq can be used in such an experiment to determine whether the progeny of a stem-like cell can give rise to all cancer cell subtypes. (*Middle*) CRISPR genome recording can be used to connect a cell’s transcriptional state at the time of analysis to its clonal ancestry. This method can be used to reveal dominant clones. (*Bottom*) CRISPR recording can also be used to record the cumulative activity of a pathway of interest. A history of pathway activity associated with increased fitness or stemness will be detected in most cancer cells. Conversely, the bulk tumor cell population will display few recorded events of a pathway that suppresses tumor progression and clonal expansion. Abbreviations: scRNA-seq, single-cell RNA sequencing; t-SNE dim *n*, *n*th dimension of t-distributed stochastic neighbor embedding.

A gold standard approach for determining the fate of a particular cellular lineage in developmental biology studies is to use lineage tracing (also known as fate mapping). In such experiments, a subset of cancer or stromal cells can be genetically labeled by a heritable genetic mark (typically a fluorescent protein). Thus, any descendants of labeled cells will inherit this genetic mark, enabling evaluation of the potential of a cell type to contribute to tumor growth or various components of the stroma (Schepers et al. 2012). This classic genetic approach offers unique advantages, particularly when combined with the contemporary armamentarium of highly multiplexed single-cell and spatial technologies (Figure 2). Such multiparameter analyses enable the identification of the full range of cellular phenotypes arising from a subpopulation of interest in unprecedented detail (Nowotschin et al. 2019).

Recent years have witnessed an explosion in the number and versatility of available lineage-tracing techniques (Spanjaard & Junker 2017). One approach involves the use of retrovirally introduced molecular barcodes that can be used to read out a cell’s clonal identity from analyzing the DNA of the cell for the integrated retrovirus (Kebschull & Zador 2018). Barcodes incorporated into expressed transcripts can be used to identify clonal relationships from scRNA-seq data (Kebschull & Zador 2018). Barcoding combined with repeated sampling and scRNA-seq was recently used to retrospectively identify cell states associated with increased fitness (Shakiba et al. 2019). A clever recent addition is the combination of barcodes with small-guide RNAs (sgRNAs) (Dixit et al. 2016) or the use of the sgRNAs themselves as cellular identifiers for CRISPR-induced mutations (Datlinger et al. 2017). These approaches combine transcriptomic information and a genetic perturbation, enabling the massively parallel phenotyping of a large pool of cells harboring a diverse set of engineered mutations. A related application is to use CRISPR for lineage tracing to introduce random mutations at a neutral site in the genome, in effect generating an evolving heritable barcode. This approach provides more dynamic information about the clonal relationships in tumors, allowing one to identify not just the trunk but also the branches of a particular cellular lineage (Figure 2) (McKenna et al. 2016, Perli et al. 2016, Spanjaard et al. 2018). Furthermore, CRISPR genomic recording can be used to quantitatively measure the activity of a particular pathway in a population of cells over time. For example, Cas9 could be placed under the control of a pathway-sensitive promoter so that recording only occurs in cells that display activity of the pathway. The clonal expansion and relationships between cells that activated the signaling pathway of interest can be tracked, but with the added information of whether some descendants of the clone reactivated the pathway at a later time during tumor progression, perhaps reinforcing the expansion of the clone (Figure 2). Finally, fluorescent markers also enable the imaging of cellular subpopulations in tumors in situ by intravital microscopy. This approach allows one to directly visualize cellular behavior, including interactions with other cells, migration, gene expression, and biochemical signaling events (Scheele et al. 2016).

A powerful parallel to lineage-tracing studies offered by GEMMs is the ability to kill a specific subset of cells in tumors to interrogate its function in tumor maintenance and progression. Such lineage-ablation experiments typically utilize a system where a suicide gene, such as diphtheria toxin receptor (DTR), is placed under the control of gene regulatory elements that specifically mark a cell type of interest. Mouse cells are not sensitive to diphtheria toxin (DT), an extremely potent cytotoxic peptide, because they do not express DTR. This enables the selective ablation of a cancer or stromal cell subpopulation of interest by systemic delivery of DT (Buch et al. 2005, de Sousa e Melo et al. 2017). In addition to suicide-gene-based approaches, certain cell types express specific surface markers that can be used to eliminate the cells using monoclonal antibodies that elicit antibody-dependent cellular cytotoxicity. This approach has been widely used to eliminate immune cells, such as CD4^+^ or CD8^+^ T cells, B cells, or natural killer cells (e.g., Cobbold et al. 1984, Daley et al. 2008, Reff et al. 1994, Seaman et al. 1987). In addition to monoclonal antibodies, macrophages can be depleted using clodronate liposomes (van Rooijen et al. 1996). Cell-ablation studies also enable one to explore the rate at which a particular cell type is regenerated, as well as to identify the cellular source from which the cell arises (Tian et al. 2011).

This combination of approaches to investigate single cells and genetic tools to model cancer and to trace cell lineages offers extremely promising avenues to track heterogeneity in tumors in mouse models.

## LESSONS AND CONCEPTS FROM THE ANALYSIS OF MOUSE MODELS OF CANCER HETEROGENEITY

The field of mouse models of cancer is vast, and an increasing number of studies have begun to interrogate cancer heterogeneity. Here we chose to discuss specific examples in which mouse models have already provided novel insights into cancer biology linked to the three types of heterogeneity discussed above (genetic, epigenetic, and microenvironmental). We have chosen to focus predom-inantly, but not exclusively, on lung cancer, which accounts for more deaths worldwide than breast, prostate, colon, kidney, and liver cancer combined; lung cancer thus continues to be a challenge for therapeutic interventions and an area of intense study. Notably, GEMMs for lung cancer provide accurate models of multiple aspects of lung cancer biology.

### Linking the Identity of the Cell of Origin to Cancer Heterogeneity in Mouse Models

Early detection of cancer is usually associated with better survival rates for patients, as small tumors are easier to resect and early-stage cancer has often not spread to other sites. For many human cancers, however, the earliest stages of tumorigenesis are challenging if not impossible to investigate. In particular, it is virtually impossible in most cases to identify the cell type from which a solid tumor arises—a notable exception includes recent studies on human retinoblastoma, which led to the identification of cone precursors in the fetal retina as a cell type of origin (Xu et al. 2014). The identification of the cell type from which cancer arises may provide some key insights into the sensitivity of cancer cells to targeted therapies (for example, if cancer cells retain similar signaling networks with the cell of origin) and may help researchers understand how tumors gain heterogeneous features, including the evolution of cancer stem cells and their progeny within tumors (reviewed by Gilbertson 2011 and Visvader 2011).

Emerging evidence in mouse models indicates that the identity of the cell of origin can impart a long-term influence on growing tumors. For example, activation of the *Kras* oncogene (*Kras*^G12D^) in different epithelial cell types in the lung epithelium, such as alveolar type II cells or bronchiolar club cells, gives rise to cancerous adenoma lesions that initially appear histologically distinct (Sutherland et al. 2014). However, eventually these lesions converge toward a similar lung adenocarcinoma histology (Sutherland et al. 2014). This study suggests that the genetic events driving cancer development are dominant and can promote a loss of epigenetic heterogeneity even when tumors start from different cell types. Such a convergence of cellular phenotypes has also been proposed in neuroendocrine cancers, including lung and prostate cancer: Similar oncogenic events (e.g., loss of the Rb and p53 tumor suppressors coupled to increased Myc activity) in normal cell types from different organs and tissues may grossly reprogram cells toward a similar cancer phenotype (Park et al. 2018).

Other studies in mouse models indicate that both the identity of the cell of origin and the initiating genetic alterations contribute to the epigenetic and genetic diversity of the resulting tumors. For instance, lung tumors initiated upon activation of Kras^G12D^ and loss of function of the Lkb1 tumor suppressor in alveolar type II cells or bronchiolar club cells in the lung epithelium have different histopathological features (Nagaraj et al. 2017). Tumors derived from club cells showed a wide spectrum of lung adenocarcinoma subtypes, with a predominance of the adenosquamous subtype. In contrast, tumors derived from alveolar cells were primarily adenocarcinomas. This heterogeneity extended to immune cells in the microenvironment, with formation of a more immunosuppressive microenvironment in the adenosquamous histotype (Nagaraj et al. 2017). Similarly, a study from the Blanpain group showed that the chromatin state of the cell of origin can dictate whether squamous cell carcinomas (SCCs) of the skin will acquire more mesenchymal features and become more metastatic (Latil et al. 2017). In this mouse model, tumors were initiated by Kras^G12D^ and loss of function of the p53 tumor suppressor. SCCs derived from the interfollicular epidermis were found to be more indolent, whereas SCCs derived from hair follicle stem cells showed more evidence of epithelial-to-mesenchymal transition (EMT) and had increased metastatic potential. Transcriptional and chromatin profiling experiments indicate that the hair follicle stem cells are epigenetically primed to undergo EMT during tumorigenesis (Latil et al. 2017). Interestingly, similar phenotypic plasticity is observed early in cutaneous SCC development, driven by the combination of epigenetic changes associated with wound healing and tumor-acquired stress factors (Ge et al. 2017).

As a third example, our recent work in small cell lung cancer (SCLC) shows that these tumors can arise from distinct cell populations in the lung epithelium (in this case, upon loss of function of the p53, Rb, and p130 tumor suppressors) (Yang et al. 2018). The tumors arising from these different lung epithelial cells become metastatic via different molecular mechanisms (Yang et al. 2018). In one case, when SCLC tumors are initiated in a non-neuroendocrine cell type, the tumors become neuroendocrine and gain metastatic ability through amplification of the gene coding for the transcription factor Nfib, which leads to an extensive remodeling of the chromatin and significant changes in gene expression programs (Denny et al. 2016). In contrast, SCLC tumors arising from neuroendocrine cells can become metastatic even in the absence of Nfib overexpression (Yang et al. 2018).

These observations of an epigenetic memory of the cell type of origin months after cancer initiation, and of different genetic mechanisms of tumorigenesis dependent on the cell type of origin, emphasize the critical need to better understand the early stages of cancer development in patients.

### Intratumoral Heterogeneity Linked to Epigenetic and Genetic Changes During Tumor Progression Toward a Metastatic State as Demonstrated in Mouse Models

The large majority of cancer patients die from metastases seeded by the primary tumor. In many cases, patients with metastases do not undergo resection of these disseminated tumors, as resection poses some risks and, in most cases, does not improve survival. This limited tissue access is one major reason why the mechanisms of metastasis remain less well understood than other mechanisms of tumorigenesis (reviewed by Lambert et al. 2017 and Nguyen & Massague 2007). A major question in the field is the nature of the events that allow cells to become metastatic: Is it possible that the genetic and epigenetic events that transform cells are also sufficient to endow these cells with metastatic potential? Or do cancer cells need additional genetic and epigenetic alterations before gaining metastatic potential? In the latter case, do these additional alterations provide some selective advantage in the primary tumor, as would be expected? When metastases have been studied from patients, some answers to these questions have been suggested. For example, mutations in p53 in prostate cancer may provide an advantage in primary tumors and also correlate with metastatic potential (Hong et al. 2015). As a second example, studies in clear-cell renal cell carcinoma identified loss of 9p as a highly selected event driving metastasis (Turajlic et al. 2018). In contrast, recent studies in human pancreatic cancer also showed that a large majority of driver gene mutations were common to all metastases studied in each patient (Reiter et al. 2018). Despite these recent findings with human tumors identifying genetic differences between primary tumors and metastases (or the absence of such genetic heterogeneity), mouse models still provide a much greater level of flexibility to collect tumor samples in a more controlled environment (reviewed by Gomez-Cuadrado et al. 2017 and Khanna & Hunter 2005).

SCLC is a disease for which little is known in terms of the mechanisms of metastatic progression. Because 60–70% of SCLC patients have metastases at the time of first diagnosis, it was assumed that SCLC cells are inherently metastatic; in other words, SCLC may represent a type of cancer in which the mutations driving the growth of primary tumors are sufficient to endow cells with metastatic potential. We isolated pure populations of cancer cells from primary tumors and metastases from a mouse model of SCLC using a fluorescent reporter specifically labeling the cancer cells. We found that not all tumors have metastatic potential. We also found a copy number amplification of the *Nfib* locus, as well as an enrichment in accessible sites for Nfib transcription factor binding in the metastases (Denny et al. 2016). Experiments in cell-based systems and mice showed that increased levels of Nfib are necessary and sufficient to promote metastasis (Denny et al. 2016, Semenova et al. 2016, Wu et al. 2016). In contrast, in a different mouse model of SCLC in which tumors are specifically induced in neuroendocrine lung epithelial cells (instead of non-neuroendocrine cells, as in the previous study), we found that *Nfib* amplification was not a frequent mechanism of metastasis (Yang et al. 2018). In humans, a majority of metastases, but not all, express high levels of NFIB (Denny et al. 2016, Semenova et al. 2016, Wu et al. 2016, Yang et al. 2018), which suggests that different mouse models may model different paths to metastatic development in humans. Genomic studies of human SCLC suggest that the *NFIB* gene is not very frequently amplified (George et al. 2015), suggesting that the switch may more often be epigenetic in human tumors (rather than genetic amplification of the locus leading to higher levels of expression). Together, these data from mouse models conclusively determine that SCLC is not necessarily inherently metastatic and identify a molecular switch in more than half of SCLC cases that endows cells with increased metastatic potential. In this context, cellular transformation is not sufficient to endow cells with metastatic potential. These studies identify a new level of intratumoral heterogeneity between primary tumors and metastases in SCLC.

In lung adenocarcinoma, similar to the SCLC models, not all mouse tumors initiated synchronously by the same activation of Kras^G12D^ and loss of p53 become malignant and metastatic, indicative of heterogeneity in tumor progression. Genomic analyses in mouse and human tumors identified the transcription factor Nkx2–1 (also known as Ttf-1) as a suppressor of metastatic progression (Winslow et al. 2011). One mechanism by which Nkx2–1 suppresses tumor progression and metastasis is by repression of the chromatin regulator Hmga2, which is normally only expressed during embryogenesis and in the adult testes (Winslow et al. 2011). While the mechanisms leading to Nkx2–1 silencing during tumor progression are not fully elucidated, deletion of the locus is rare (Winslow et al. 2011), suggestive of epigenetic silencing mechanisms such as DNA methylation. Interestingly, the Nkx2–1 target Selenbp1 (selenium-binding protein 1) was shown to be both an important regulator of metastatic progression and a regulator of Nkx2–1 expression, in a positive feedback loop (Caswell et al. 2018). During lung adenocarcinoma progression, a switch in metastasis between short and long isoform expression of the invadopodia scaffold protein Tks5 provides another example of functional nongenetic heterogeneity during metastatic progression (Li et al. 2013). Further comparison between primary tumors and metastases in the same mouse model of lung adenocarcinoma driven by activation of Kras^G12D^ and loss of p53 also identified the basic helix-loop-helix transcription factor Arntl2, which controls a prometastatic secretome (Brady et al. 2016). The mechanisms leading to Arntl2 induction during the metastatic process are still unclear but may be in some ways related to Nkx2–1 silencing (Brady et al. 2016). One of the targets of Arntl2 is the extracellular matrix-associated protein Smoc2, whose induction may help create a microenvironment conducive to lung adenocarcinoma metastasis (Brady et al. 2016). A new tumor barcoding system in the same mouse model allowed the Winslow lab to distinguish nonmetastatic primary tumors from primary tumors that had formed macrometastases. RNA-seq comparing these two types of primary tumors as well as metastases helped identify Jak (Janus kinase)/Stat (signal transducer and activator of transcription) signaling as an important driver of metastatic progression downstream of the CD109 cell surface receptor (Chuang et al. 2017). In these models, cellular transformation was again not sufficient to endow cells with metastatic potential. In all these different examples, analysis of human lung adenocarcinoma samples confirmed modulation of these genes and pathways in subsets of human tumors.

Sophisticated GEMMs of human pancreatic ductal adenocarcinoma (PDAC) have also been developed. In these models, Kras is activated and p53 is mutated by using a Cre-Lox strategy, where Cre is expressed under a pancreatic epithelial cell-specific *Pdx1* or *Ptf1a* promoter. These mice develop tumors that share several key features of human tumors, including stage-wise progression from pancreatic intraepithelial neoplasia (PanIN) to full-blown PDAC, as well as the development of a fibrous, desmoplastic stroma that hinders drug delivery and impairs the infiltration of cytotoxic T lymphocytes (Hingorani et al. 2005, Lee et al. 2016, Qiu & Su 2013). PDAC in this model is characterized by considerable intratumoral heterogeneity. In an interesting manifestation of this heterogeneity, a subset of PanIN cells undergo EMT and enter the blood circulation early in tumor development (Rhim et al. 2012). Subsequent work has indicated that EMT can occur by two distinct programs in PDAC tumors, such that the early PanIN cells undergo partial EMT, and the full program is only detected in tumors that progressed to PDAC, executed only by a subset of cells (Aiello et al. 2018). The transition from the PanIN stage to PDAC is associated with loss of p53 function in both human and mouse PDAC. Muzumdar et al. (2016) studied this process using mosaic analysis with double markers, where a sporadic Cre-induced p53 loss-of-function event leads to the acquisition of a fluorescent color. The p53 loss-of-function mutants expanded rapidly, suggesting that p53 constrains the progression of Kras-mutant PDAC tumors (Muzumdar et al. 2016).

Thus, the ability to track and isolate pure populations of cancer cells and the comparative analysis of primary tumors and their metastases in mouse models can help identify key genetic and epigenetic changes during tumor progression that control the metastatic spread of lung cancer cells. These experiments define several levels of intra- and intertumoral heterogeneity that are difficult to identify in cancer patients.

### Uncovering Signaling Mechanisms Driving the Generation of Epigenetic Intratumoral Heterogeneity from Cancer Stem Cells in Genetically Engineered Mice

An appealing model of tumor growth is based on cancer stem cells (also called tumor-initiating cells, tumor-reinitiating cells, or tumor-propagating cells). In this model, cancer is a hierarchical structure in which a subset of cancer cells are critical for the long-term expansion of a tumor; through their ability to give rise to differentiated progeny, these cancer stem cells, like stem cells in normal tissues, are also responsible for the cellular heterogeneity of tumors (reviewed by Batlle & Clevers 2017, Clarke et al. 2006, Magee et al. 2012, and Visvader & Lindeman 2012). Not all tumors appear to rely on this hierarchy, for example in cases where cancer stem cells are very frequent (Meacham & Morrison 2013, Quintana et al. 2010). It is also sometimes difficult to tease apart heterogeneity related to epigenetic differences (following the cancer stem cell model) and heterogeneity related to genetic differences (following a clonal evolution model) (Shackleton et al. 2009, van Niekerk et al. 2017). As with other aspects of tumorigenesis, mouse models can help clarify these issues, providing a genetically controlled environment as well as appropriate lineage-tracing and lineage-ablation tools.

**Figure 3:**
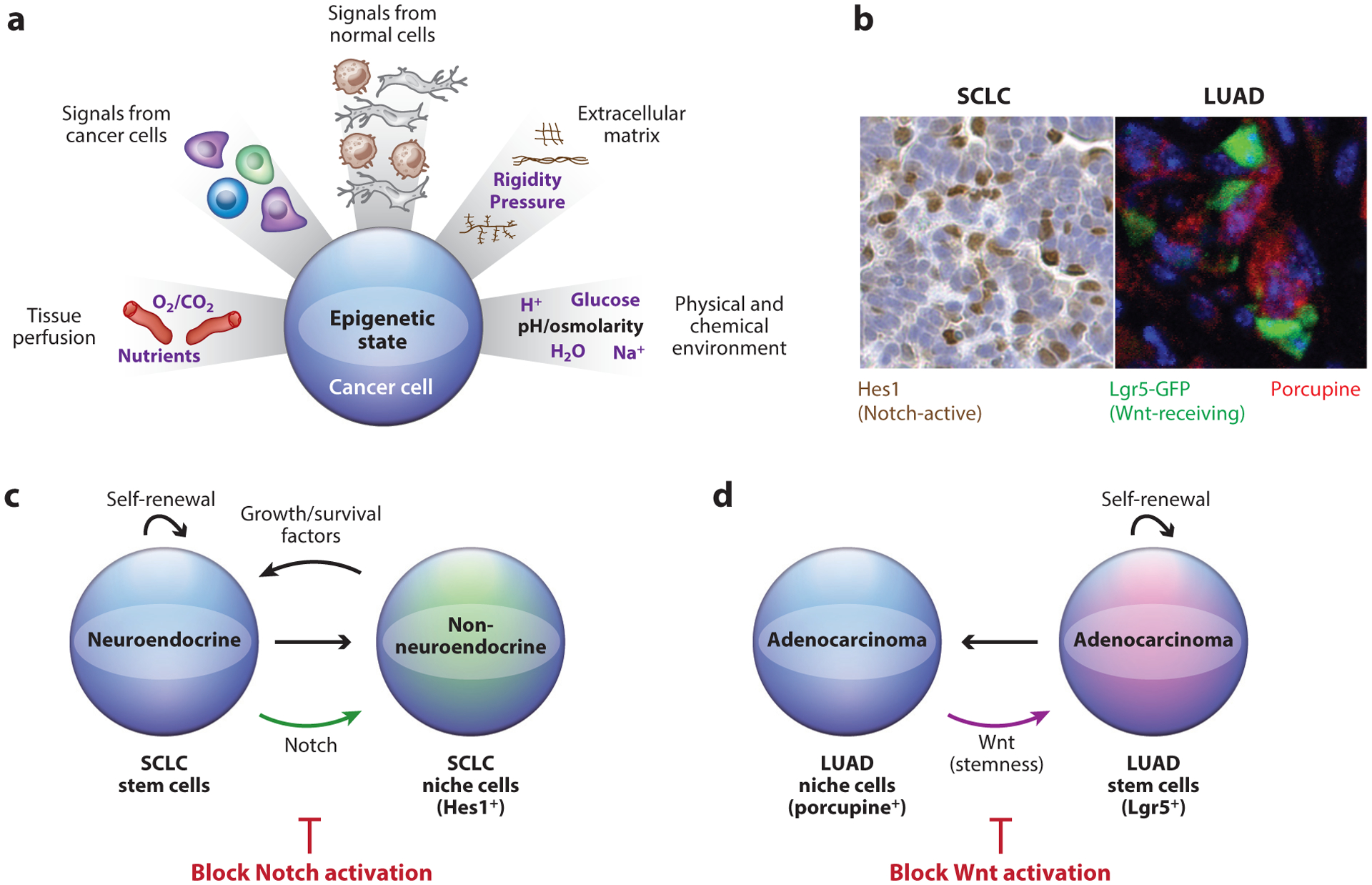
Cell-extrinsic signals that can generate epigenetic heterogeneity in cancer cells. (*a*) The epigenetic state of cancer cells is under the control of multiple factors in the microenvironment. (*b*) Representative images of heterogeneity in mouse models of small cell lung carcinoma (SCLC) and lung adenocarcinoma (LUAD). (*Left*) Immunohistochemistry (*brown*) for Hes1 marks the nucleus of Notch-active SCLC cells in a *p53/Rb/p130*-mutant mouse. The counterstain is hematoxylin (*purple*). (*Right*) Immunostaining for green fluorescent protein (GFP; *green*) and porcupine (*red*) in a subcutaneous transplant of primary *Kras*^G12D/+^*;p53*^Δ/Δ^*;Lgr5*^GFP-CreER/+^ mouse LUAD cells three weeks after transplantation. DNA is stained in blue. Note the juxtaposition between the green GFP^+^ Lgr5^+^ cells and the red porcupine^+^ cells. SCLC image courtesy of the Sage lab; LUAD image reproduced with permission from Tammela et al. (2017). (*c*) In SCLC, some of the cancer stem cells change their fate upon activation of Notch signaling: The newly formed non-neuroendocrine cancer cells (Hes1^+^) serve as a supportive niche that produces growth and survival factors. Blocking Notch activation may be a therapeutic strategy to prevent the generation of the supportive niche population. (*d*) In LUAD, cancer stem cells rely on Wnt signaling for long-term expansion. Some of these stem-like cancer cells (Lgr5^+^) can differentiate into a supportive niche population that secretes active Wnt ligands (porcupine^+^), thereby allowing the maintenance of the reciprocal interaction. Blocking the generation of active Wnt ligands or Wnt signaling may be a therapeutic strategy to prevent the long-term expansion of LUAD stem cells.

For example, the aggressive nature of SCLC may suggest that these tumors do not follow the hierarchical cancer stem cell model—perhaps all types of cancer cells can propagate tumors in the long term? However, it has been known for a long time that SCLC cell populations show heterogeneity in the expression of cell surface receptors (Fargion et al. 1986). Indeed, using a genetic model of SCLC and transplantation assays, we found that approximately half of the cells in mouse SCLC tumors could transplant new tumors. These cells maintained their neuroendocrine features (including robust expression of NCAM on their surfaces), epithelial identity (robust expression of EpCAM on their surfaces), and expression of high levels of CD24 and low levels of CD44 (Jahchan et al. 2016). The same cell surface markers were found on human SCLC cells. While it is possible that additional markers may further define cancer stem cells in SCLC, this analysis of a mouse model helped define the first major subpopulation of SCLC cells. Furthermore, these SCLC cancer stem cells express cell surface markers that can be targeted for therapy, including the atypical Notch ligand Dll3 (Rudin et al. 2017, Saunders et al. 2015) and NCAM (Socinski et al. 2017). Other subpopulations of SCLC cells have been identified in human tumors [e.g., cancer cells differentiating toward a vascular phenotype (Williamson et al. 2016)] and in mouse models (Calbo et al. 2011, Kwon et al. 2015, Lim et al. 2017). First, Anton Berns’s group identified a population of non-neuroendocrine SCLC cells expressing mesenchymal markers such as vimentin (Calbo et al. 2011). Importantly, these cancer cells, which express high levels of CD44, can enhance the ability of other SCLC cells to seed new tumors via paracrine mechanisms involving FGF2 (Kwon et al. 2015). It is unknown if similar cells have the same tumorigenic and prometastatic potential in human SCLC, but these data provided the first proof-of-concept that tumor heterogeneity in SCLC may promote tumor progression. In support of this concept, we have identified a role for Notch signaling in SCLC growth and response to cellular stress. While Notch signaling is inherently tumor suppressive in SCLC (George et al. 2015), we found in a mouse model that several SCLC cells within tumors activate the Notch pathway. These Notch-active SCLC cells lose their neuroendocrine features, but they appear to be distinct from the Cd44^high^ mesenchymal SCLC cells (Lim et al. 2017). Importantly, these Notch-active SCLC cells also functionally interact with the neuroendocrine cancer cells and promote their survival and expansion, including following treatment with chemotherapeutic agents (Figure 3). Thus, while SCLC tumors are notorious for including few noncancer cells, mesenchymal SCLC cells, vascular-like SCLC cells, and Notch-active SCLC cells may all contribute to tumor development, metastasis, and response to treatment. One aspect of these studies that remains unclear is the connection between SCLC cancer stem cells, chemoresistance, and the acquisition of metastatic potential.

Stem cells have the capacity to self-renew while also producing differentiated cells. In normal tissues, these choices are primarily dictated by extrinsic signaling factors, which, together with the cells that produce them, comprise a niche with a short range of action, which limits the number of stem cells. Among the niche signals that promote stem cell phenotypes, secreted Wnt proteins are notable due to their function in multiple stem cell compartments. The Wnt target gene *Lgr5* is specifically expressed in stem cells in various epithelial tissues (Barker et al. 2007, Jaks et al. 2008). Lineage tracing using a multicolor Cre recombinase Confetti reporter system was used to demonstrate that Lgr5 marks stem-like cells in colon adenomas (Schepers et al. 2012). Conversely, differentiated colon cancer cells marked by Krt20 expression were postmitotic and demonstrated very low potential for clonal expansion (Shimokawa et al. 2017). In colon adenomas and adenocarcinomas, the ablation of the Lgr5^+^ cell subset suppressed tumor growth and eradicated liver metastases (de Sousa e Melo et al. 2017). Interestingly, in conditions where the Lgr5^+^ cells were depleted, a subset of the Krt20^+^/Lgr5^−^ colon cancer cells were capable of reverting to Lgr5^+^ stem-like cells by entering the niche vacated by the ablated Lgr5^+^ cells (Shimokawa et al. 2017). These findings suggest that carcinoma cells can be remarkably plastic and highlight the importance of local signaling microenvironments in shaping cellular phenotypes.

Signals that induce and maintain stem-like cells in cancer can present useful targets for therapeutically targeting tumor heterogeneity. Using a lung adenocarcinoma model driven by activation of Kras^G12D^ and loss of p53, we demonstrated that these tumors showed hierarchical features with two distinct cancer cell subpopulations: one with increased Wnt signaling activity, and another forming a niche that provides the Wnt signal (Tammela et al. 2017). We found that the niche cells are marked by porcupine, an enzyme that performs an essential lipid modification of Wnt ligands (Figure 3). The cells in close proximity to the Wnt signaling centers displayed increased proliferative potential, as demonstrated by lineage tracing. Inhibiting Wnt ligand production with small-molecule porcupine inhibitors reduced the proliferative potential of tumor cells, which translated into increased animal survival. Interestingly, the therapeutic effect of porcupine inhibitors appeared to arise from a reduction in proliferative potential within the lung cancer cell population, suggesting loss-of-stemness-related features. Subsequently, we identified similar niches and cells with high-Wnt pathway activity in colorectal cancers and in pancreatic adenocarcinomas, implicating that similar mechanisms may be in play in other types of cancers (Tammela et al. 2017).

### Tumor Heterogeneity and Response to Therapy

**Figure 4:**
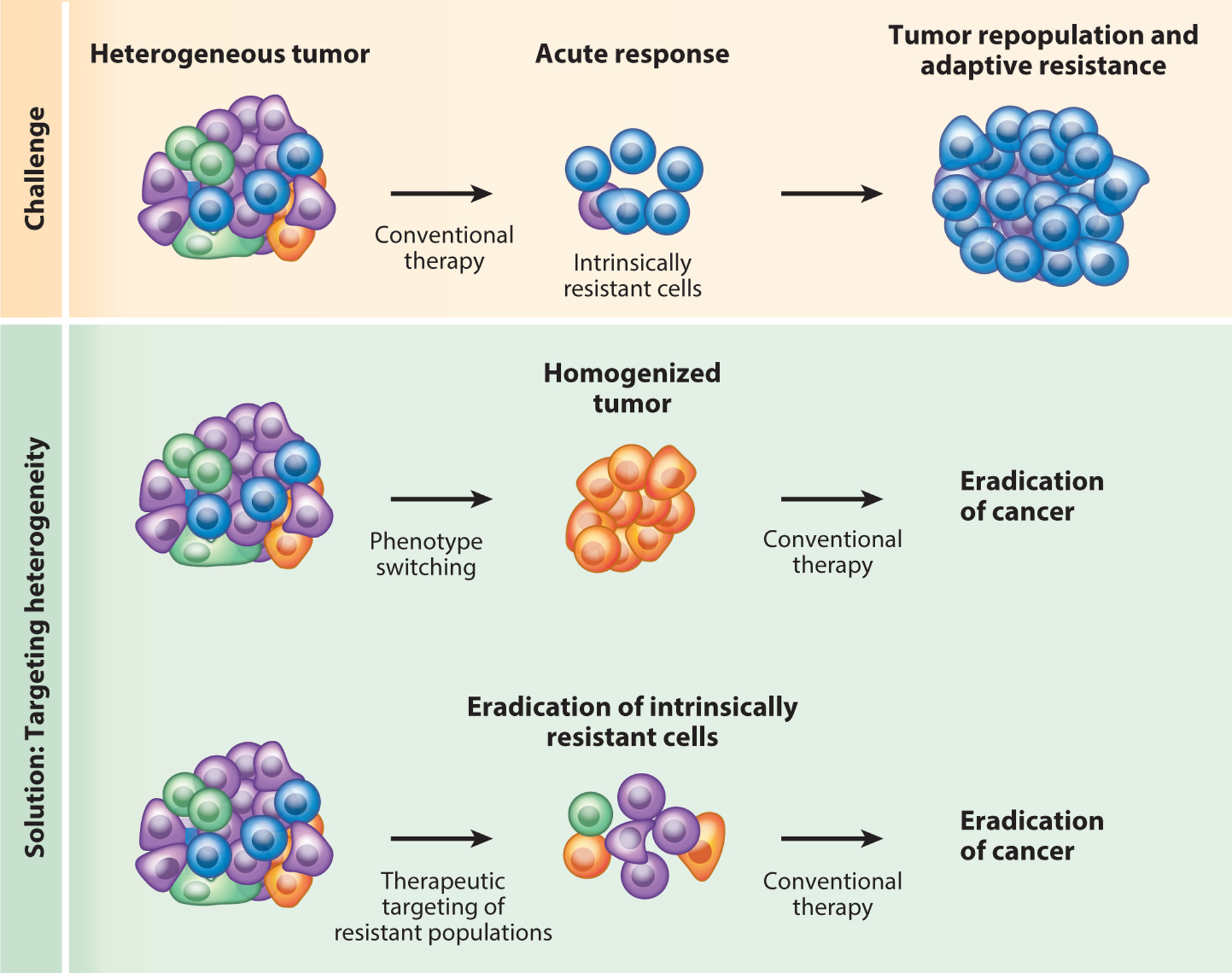
Strategies to target heterogeneity in cancer. (*Top*) Tumors contain heterogeneous subpopulations of cancer cells that are intrinsically resistant to therapies, enabling the tumor to acutely evade therapy and develop adaptive resistance. (*Bottom*) Targeting mechanisms that drive heterogeneity can either push cancer cells into states that are responsive to conventional therapies (phenotype switching) or eliminate specific subpopulations through the identification of critical druggable dependencies before or during treatment of the tumor with conventional therapy.

The diversity of cellular states within tumors poses a challenge for effective cancer treatments: Cancer cells capable of withstanding and adapting to therapies invariably exist. The identification and therapeutic manipulation of such intrinsically resistant cellular subtypes can lead to improved outcomes (Figure 4). For example, secreted Wnt signals maintain a subpopulation of skin basal cell carcinoma cells in a stem-like state, which protects these cells from hedgehog signaling inhibitors, enabling them to initiate tumor relapse upon withdrawal of the inhibitors (Sanchez-Danes et al. 2018). As such, the combination of hedgehog inhibitors with porcupine inhibitors effectively suppressed relapse, producing complete responses in a majority of tumors (Sanchez-Danes et al. 2018). Another example of such a treatment-resistant subpopulation are Nestin^+^ glioblastoma stem cells (Chen et al. 2012). These cells are quiescent and highly resistant to temo-zolomide chemotherapy, but rapidly engage the cell cycle and repopulate the tumors following cessation of therapy. Interestingly, the experimental ablation of the Nestin^+^ glioblastoma cells in combination with chemotherapy resulted in an inhibition of glioma progression in the cerebra of treated mice (Chen et al. 2012).

These studies highlight the power of mouse models in enabling discoveries with profound translational potential that would be impossible in human patients. Future studies will continue to leverage these models to improve the design of therapies in patients and to enable identification of fundamental mechanisms of tumorigenesis.

## FUTURE ISSUES

1. The generation of new mouse models for the functional interrogation of cancer cell subpopulations is a major goal in the field. Mouse models are needed that enable the lineage tracing, lineage ablation, imaging, and isolation of candidate cancer cell subpopulations. Such models will greatly further our understanding of the interdependencies between cancer cell states and the molecular mechanisms (transcriptional programs and signaling pathways) that support these various cell states.
2. Uncovering mechanisms that enable cellular plasticity should remain an important goal. Cellular plasticity poses a tremendous obstacle for effective therapies. Epigenetically plastic cells are capable of dramatic phenotypic transformations, such as epithelial-mesenchymal transition or lineage switching—as exemplified by the transformation of prostate or lung adenocarcinomas into neuroendocrine tumors under therapeutic pressure (Ku et al. 2017, Mu et al. 2017, Niederst et al. 2015, Sequist et al. 2011). The molecular events enabling lineage plasticity are poorly understood.
3. The molecular definition of cell states needs to be refined. Current methods enable the characterization of cell states in exquisite detail at single-cell resolution. However, the integration of genomic, transcriptional, proteomic, and metabolomic information is needed to fully appreciate cell states. Such data integration would be extremely powerful in generating testable hypotheses to identify mechanisms that drive cell state heterogeneity.
4. Developing our knowledge of cellular ancestry and clonal evolution is critical for understanding tumorigenesis. The current panorama of approaches used to define cellular heterogeneity is becoming increasingly sophisticated. However, most of the work in the field is done by analyzing the same tumor at single time points. Thus, the multitude of genetic, epigenetic, and signaling events that produced a particular cell state remains a mystery. Methods are needed that enable the recording of such events into a cell’s DNA, the prospective identification of cells likely to undergo a particular event, and the longitudinal tracking of clonal behavior.
5. The interrogation of cellular microenvironments is central to our understanding of the tumor milieu. It is becoming increasingly clear that the local tissue microenvironment is a central player in driving cell state heterogeneity in tumors. Developing our understanding of the spatial context in which cells reside at the molecular and cellular level will lead to critical insights into understanding cellular diversity in tumors.
6. Understanding how therapy shapes tumor heterogeneity is a crucial issue in developing new treatment options in patients. Cancer therapies exert a selective pressure on cancer cells, enabling those fittest to survive to repopulate the tumor with treatment-resistant cells. More information is needed on what defines intrinsic drug resistance and the events that lead to tumor outgrowth from minimal residual disease.
